# Perceived Mortality and Perceived Morality: Perceptions of Value-Orientation Are More Likely When a Decision Is Preceded by a Mortality Reminder

**DOI:** 10.3389/fpsyg.2016.00233

**Published:** 2016-03-01

**Authors:** Mads Nordmo, Elisabeth Norman

**Affiliations:** ^1^Department of Strategy and Management, The Norwegian School of EconomicsBergen, Norway; ^2^Department of Psychosocial Science, Faculty of Psychology, University of BergenBergen, Norway

**Keywords:** mortality salience, terror management, CSR communication, attribution, value-orientation

## Abstract

The questions addressed in this paper are whether and how reported mortality reminders can function as an indication of sincerity when communicating ambiguously motivated decisions. In two experiments, participants were exposed to a fictitious CEO who announced a decision to implement new organizational measures that were both environmentally and financially beneficial. In the experimental condition, the CEO attributed her new ideas to a recent mortality reminder. In the active control condition, the CEO attributed her decision to a non-lethal dentistry health scare, and in the passive control condition the CEO did not give any account of events preceding her decision. When a CEO implemented new corporate initiatives after a mortality reminder, her motivation for doing so was perceived as somewhat more motivated by intrinsic values, and significantly less motivated by financial gains. This change in attribution patterns was demonstrated to be indirectly related to a positive evaluation of the CEO, as well as an increased willingness to pay for the organization’s services. The second experiment further demonstrated that the reduced attribution to financial motivation associated with mortality awareness persisted even when the CEO in question was known for placing a high personal priority on financial goal attainment. The findings underscore the importance of perceived value-oriented motivation when communicating climate change mitigating policies, and the role of mortality awareness as one of many ways to induce such attributions.

## Introduction

There is a growing consensus that corporations have a social responsibility to serve communities, society, and the environment in ways that go above and beyond what is legally required (Wood, 1991; McWilliams and Siegel, 2001; Lockett et al., 2006). This form of social responsibility is typically referred to as *Corporate Social Responsibility* (CSR). The goal of most CSR initiatives is to achieve sustained competitive advantage by attracting and retaining support from consumers and other stakeholders (Waddock, 2008; Devinney, 2009; Porter and Kramer, 2011). CSR measures frequently involve activities and goals that appear to go against the corporate logic of profit, such as philanthropy, community development, environmental conservation, or social justice (Van Marrewijk, 2003). In many cases, however, the CSR goals neatly overlap with the ordinary corporate goals, such as reducing costly waste and conserving energy.

Organizations and individuals face a dilemma regarding how to communicate their CSR policies to the public. In general, most consumers want corporations to act as good corporate citizens. However, consumers are often also quite skeptical of corporations that promote their good citizenship (Brønn and Vrioni, 2001; Morsing et al., 2008; Lii and Lee, 2012). Yoon et al. (2006) found that in cases where a company was perceived as insincere, CSR communications actually hurt the company image. Similarly, psychological research has demonstrated that perceived intentions and motivations are crucial when labeling actions morally good or bad. For a behavior to be considered morally praiseworthy, the agent must not only have intended and brought about the action and its consequences, she must also have performed the act for reasons that are themselves praiseworthy (Fedotova et al., 2011; Gray et al., 2012; Critcher et al., in preparation). Organizations thus face a perilous and delicate situation. By under-communicating CSR activities, one faces the risk of people never learning about the activities, and possibly assuming that no CSR initiatives have been made. By failing to communicate CSR messages in a sincere and believable fashion, one faces the risk of skepticism and cynicism among of weary consumers, who disbelieve the accuracy and sincerity of the claims, and the efficacy of the policies (Morsing et al., 2008). This danger of perceived insincerity is also relevant when corporations engage in environmentally friendly activities. Even though communicating environmentally oriented CSR policies and activities could elicit positive reactions, it may also lead to adverse motivational attributions (see Groza et al., 2011). In cases where the sustainability initiatives are seen as primarily motivated by financial gain, people often respond negatively (Morsing and Schultz, 2006). Previous research has demonstrated that people easily suspect ulterior motives when they hear of corporate activities that are both environmentally and financially beneficial (de Vries et al., 2013; Windsor, 2013). As perceived sincerity of motivation has been identified as a key success factor for CSR communication, and as CSR is becoming an increasingly important part of brand strategy, it is important to understand how consumers perceive the motivation behind different CSR strategies, and identify the antecedents to favorable motivational attribution (Du et al., 2010; Debeljak et al., 2011; Olsen et al., 2014).

In this paper, we explored how justifying ambiguously motivated CSR initiatives with a recent mortality reminder can mitigate suspicion of extrinsic motivation. The overarching research question was whether people recently exposed to a mortality reminder are perceived as more value-oriented. By perceived value-orientation, we mean a combination of more attribution to intrinsic moral or ideological convictions, and less attribution to extrinsic factors, such as financial gains, and external expectations on behalf of consumers and stakeholders. The research question is particularly interesting in order to understand how people perceive individuals and organizations who attempt to portray themselves as morally motivated, while obviously also being motivated by financial gain. It is also of immediate concern for research on how to increase favorable evaluation of climate change mitigating CSR initiatives in the population at large, as cynical motivational attribution may be a barrier against positive evaluations of such initiatives. While previous research has speculated that mortality awareness can aid people into more environmentally friendly attitudes and behaviors (Vail et al., 2012), we extend the investigation into how a motivational attribution can be altered when we know that the observed person is in a state of elevated mortality awareness.

### Mortality Salience

The psychological and behavioral consequences of thinking about death, and the mortality of the self and others, have been studied extensively (Burke et al., 2010). Most of this research has been conducted within the framework of terror management theory (Greenberg et al., 1997; Pyszczynski et al., 1999). Typical terror management has been studied in experiments where one group is exposed to a mortality prime, e.g., an instruction to describe what happens to the physical body after death, while a control group is exposed to a control prime, e.g., an instruction to describe a painful dentistry treatment (Burke et al., 2010). After an initial priming manipulation, participants then go on to indicate their attitudes, or perform behaviors that are hypothesized to be affected by the presence of mortality awareness. According to TMT, people have an innate existential motivation to turn to meaning-providing structures to cope with the knowledge of inevitable mortality (Greenberg et al., 1990; Routledge et al., 2008). The most commonly observed coping mechanisms are increased investments in-, and defense of, own cultural world views, and self-esteem (Baumeister, 1991). Subsequent increase in self-esteem and an increased defense of one’s own cultural world views have been observed as a reaction to mortality primes in a vast number of studies (Burke et al., 2010), while a slight shift toward political conservatism has been observed in others (Jost et al., 2003). These reactions are typically referred to as ‘distal defenses’, meaning that the process by which they arise as a response to the mortality prime is typically not consciously accessible (Greenberg et al., 2000). Terror management studies using attributions as outcome measure have yielded findings in line with the theoretical framework, demonstrating that mortality primes increase the tendency for self serving attributions and imbuing everyday actions with meaning (Mikulincer and Florian, 2002; Landau et al., 2010).

### Mortality Salience and Different Motivations

The idea that awareness of one’s own mortality may be related to changes in the priority of extrinsic pursuits is not new. All five major religions present recurrent reminders of how material riches are rendered empty in the face of mortality. Works of literature and philosophy further echo the notion that the thought of one’s inevitable death can make efforts to obtain superfluous material value seem inauthentic and meaningless. The existential psychologists Yalom (1980, 2008), as well as classic literary characters like Dickens’s Ebenezer Scrooge, have illustrated how intimations of mortality make strivings for wealth and social status seem vacuous and void of meaning. Councilors working with terminal patients, or people who have had near-death experiences, report that a typical reaction is to devaluate the meaning of material possessions and ego-enhancement (Kinnier et al., 2001; Ware, 2011). The effects of mortality primes on materialism and extrinsic vs. value-driven motivation have been researched quite extensively. While philosophy and literature point solely toward a decrease in extrinsic motivation as consequence of mortality reminders, TMT experiments offer more complex results. Kosloff and Greenberg (2009) found that participants who were asked how much importance they placed on extrinsic pursuits tended to trivialize their importance if asked directly after the mortality prime. However, when given a distractor task between the presentation of the mortality prime and the subsequent questionnaire, participants gave higher importance ratings for a high priority extrinsic goal. The authors argued that such effects may arise because the affirmation of personally important extrinsic goals can lead to higher self-esteem and defense of the sources of meaning in life. Across most TMT experiments, the delay and distraction between the mortality prime and outcome measure is used to allow for mortality cognitions to fade from consciousness, as the distal defenses are theorized to only manifest after the thought has faded from consciousness (Burke et al., 2010). Increase in self-esteem and embracement of one’s cultural world views are the two well-known distal defenses, and as such, it is in line with TMT that the increased investment in extrinsic goals after a mortality prime is only present when a distractor task is used, and the extrinsic goal considered culturally and/or personally important (Arndt et al., 2004). Support for pro-environmental attitudes can be increased as a reaction to a mortality prime, but only in cases where people are already imbued with pro-environmental attitudes (Vess and Arndt, 2008; Fritsche and Häfner, 2012), or when pro-environmental norms are a salient part of the environment (Fritsche et al., 2010). Taken together, these findings suggest that the predictable reaction toward a mortality prime is increased defense of one’s cultural worldview and self-esteem. Provided that extrinsic pursuits are the central theme in one’s cultural worldview, and/or the prominent source of positive self-esteem, mortality primes should induce an increased investment in those extrinsic pursuits. If morally motivated pro-environmentalism is an important part of one’s cultural world view, and/or important source of self-esteem, mortality primes should reliably induce increased engagement in those pursuits.

While some research has focused on the link between mortality salience, extrinsic motivation, and environmental attitudes and behavior, less is known about how mortality salience can affect the motivational attribution of other people’s behavior. Even though the presented paper draws upon TMT research, it departs from the terror management tradition in one crucial aspect. Whereas most TMT research focuses on how people respond when primed with reminders of their own mortality, this study explores how an observed decision maker is perceived, when that decision maker claims to have been made more acutely aware of her own mortality. To the very best of our knowledge, people’s lay-theories about the nature of motivation under mortality awareness have not previously been described in the literature. However, past research has demonstrated that people often infer that the motivational processes they experience are present in the minds of others as well (Reeder and Trafimow, 2005). This is why we chose to base the direction of our hypotheses on TMT research. The experiments presented here were designed to test if and how a decision makers’ claimed mortality awareness can eschew motivational attribution from financial and extrinsic to value-oriented. The experiments tested perceptions of behaviors and decisions made by an executive in a corporate setting.

### Research Outline: Mortality Awareness and Perceived Value-Orientation

The independent variable (IV) in TMT research is the presence or absence of a psychological prime that makes the issue of mortality more or less salient in the experiment situation. As the present research in more concerned with social perception (i.e., the perception of others), the IV in this study was termed morality awareness, referring to the extent to which the decision maker claimed to have been acutely aware of her own mortality. According to Critcher et al. (in preparation), people engage in social-cognitive mind reading when assessing how morally praiseworthy an observed behavior is. This mind reading entails picking up cues that indicate the extent to which the observed person seems to appreciate the underlying moral principle behind their behavior. We hypothesized that mortality awareness may serve as such a cue, highlighting that the demonstrated behavior is more motivated by moral values such as a sincere appreciation for the importance of sustainability and environmental protection, and less by financial gains and external expectations. The setting of the experiment was a description of a fictitious company that had recently decided to implement CSR measures that were not only environmentally beneficial, but also cost saving for the company, as the customers would bear the cost of the initiatives. In this particular case, the setting is a hotel chain manager who decided to reduce the size of plates and glasses at the buffet, install water saving showers, and adopt a more restrictive policy regarding changing of towels and linens. These initiatives are all clearly environmentally friendly, as they will lead to reduced waste of food and drink, reduced energy consumption, and reduced use of detergents and emission of wastewater. They are also certainly financially beneficial, as they will reduce costs on behalf of the hotel. The CEO in question claimed that the new initiatives were motivated by her sincere appreciation for the importance of environmental care and climate change mitigation. However, as all these cost-saving measures are ultimately carried by the consumer, and directly and positively influencing the company cash flow, the sincerity of the CEO’s motivation was clearly questionable. Our first prediction, derived from TMT, was that the CEO who decided to implement these measures would be presumed to be more motivated by her intrinsic values, and less motivated by extrinsic factors, if she was perceived as having been acutely aware of her own mortality when she made the decision. Our second prediction was that this relationship would be reversed, if the observed decision maker was known to put a strong personal priority on financial goal attainment. Two experiments were designed to test each of the predictions, respectively.

## Experiment 1

Experiment 1 was designed to explore if a person’s ambiguously motivated decision is attributed to different motivations, depending on whether, or not the decision maker has recently been reminded of her own mortality. The IV was the circumstances that led the CEO to come up with the new initiatives. The three experimental conditions only differed in how the CEO answered to a question of how these ideas came about, one of which involves mortality awareness. In the mortality awareness condition, the CEO claimed to have had a mortality reminder, and subsequently decided to implement the aforementioned CSR initiatives. We predicted that this set of events would lead to more value-based attributions, and less attributions to extrinsic factors, compared to the control conditions, which do not involve mortality awareness. As value-based attributions were expected to be associated with support for CSR initiatives, the first hypothesis predicted a direct effect between mortality awareness as justification, and favorable evaluation of the CSR initiatives.

H1: Reporting that a mortality reminder preceded the decision to implement environmentally friendly policies will lead to (a) more positive evaluation of decision maker and (b) higher willingness to pay, compared to active, and passive control.

We further predicted that the participants would expect a decision maker who was highly aware of her own mortality to be more inclined to make decisions that were motivated by her ideology, morality and values, compared to a decision maker who did not come across as acutely aware of her own mortality. More specifically, we predicted that the mortality awareness would lead to an increase in value-based motivational attribution, and reduction in attribution to extrinsic motivations. The second hypothesis was thus:

H2: Reporting that a mortality reminder preceded the decision to implement ambiguously motivated policies will lead to (a) more attribution to value-based motivation, and (b) less attribution to extrinsic factors, compared to both control conditions.

Past research in moral psychology has demonstrated that perceptions of underlying intentions are crucial in determining whether or not a behavior is morally praiseworthy (Fedotova et al., 2011; Gray et al., 2012), and CSR research has demonstrated that perceived sincerity of motivation is a crucial success factor for CSR communication (Du et al., 2010; de Vries et al., 2013). Our final prediction was therefore that the positive perceptual outcomes generated by using mortality reminder as justification would be mediated by a higher tendency to attribute the measures to value-based motivation. The third and final hypothesis was therefore:

H3: The relationship between presence of mortality awareness in justification and (a) more positive evaluation of decision maker and (b) higher willingness to pay, will be mediated by degree of value-based attributions.

**FIGURE 1:**
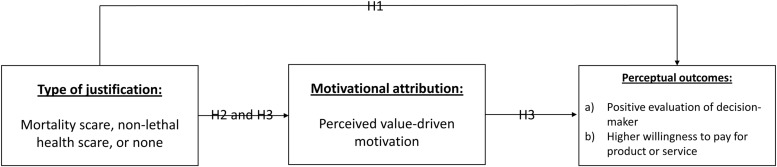
**Conceptual model**.

**Figure 1** displays the conceptual model for Experiment 1, with predicted paths in accordance with the hypotheses.

### Materials and Pre-Test

Both experiments used video footage of an actress portraying a CEO in an interview setting. The actress was instructed to portray her character in a realistic manner, while maintain identical postures, facial expressions, and tone of voice in all recordings. The video started with a rolling text stating: *“Ellen Hansen is the CEO of a large Nordic hotel chain. She has just approved a new plan to make the hotels more environmentally friendly. Among the measures are reducing the size of plates and glasses at the breakfast buffet with 20 percent, in order to reduce waste of food. Furthermore, the hotels have been equipped with water-saving showers. Additionally, the hotels have adopted a more restrictive policy on changing of towels and linens on shorter stays.* [New paragraph] *In an interview, Ellen explained the background for the decision:*” Here, the video continued to a fictitious interview, wherein the actress portraying the CEO, stated: *“We are now taking measures in order to become more socially responsible. The climate threat is one of the biggest challenges that humanity has ever faced, and our chain has to be a part of the solution”*. This part of the video constituted the passive control condition, and was identical for all conditions. The independent variable, i.e., the justification for the decision, was introduced at the end of this video. The screen showed the text: *“When asked how she got the idea for these measures, she replied:*”. The mortality awareness video showed that the CEO attributed her decision to a recent mortality reminder: “*A while ago I discovered a lump in my armpit. I contacted the doctor, who informed me that he couldn’t say for certain what this was, but that it could be the early form of a lethal and incurable form of cancer. They took a sample of the cells, and sent it to a lab for analysis. I had to wait 2 weeks for the results to arrive. The waiting was very demanding, and it got me thinking about what really matters in life. That’s when I decided to run the company in a more sustainable direction. The results came back negative, and the lump disappeared, but the motivation stayed with me*”. In accordance with past TMT research (see Burke et al., 2010), the active control video displayed the same CEO, who attributed her decision to a recent non-lethal dental health scare: “*A while ago I got a terrible oral infection. I contacted the dentist, who told me that it was either something that would pass away by itself, or an infection of the gums, in which case I would have to undergo a harmless but painful operation. He took some samples, and told me to wait a couple of weeks for the results. The waiting period was very demanding, and it got me thinking about what really matters in life. That’s when I decided to run the company in a more sustainable direction. The results came back negative, and the pain went away, but the motivation stayed with me*”. This active control condition contained many of the same elements as the mortality awareness condition, in that they both entailed insecurity, loss of control, physical pain, and personal health problems. Both conditions were liable to induce pity on behalf of the observer, and both could be seen as candid and forthright accounts of a private matter. The significant difference between the experiment condition and the active control condition was the mortality reminder referenced in the experiment condition, pitted against the explicit non-lethality of the dentistry condition.

Before the experiments, all video stimuli was pre-tested on a student sample. A total of 11 participants saw both versions of the video, in random order, producing a total of 22 observations. The pre-test examined how the actress was perceived in each video in terms of how enthusiastic and interpersonally warm the CEO was rated. Across the observations, no significant differences between the groups were found. After the test, the participants were told how the stimuli were to be used, and asked if any of the videos stood out as different from the other, aside from the different words the actress conveyed. None of the participants indicated that any of the videos differed from the others in such a way.

### Sample and Procedure

Participants (*N* = 87) were recruited from a Norwegian university law school (40 female, mean age 22). Participation was compensated with a 60 NOK (∼11 $) gift card to the student cafeteria. Before the experiment, participants were told that the experiment would be about communication, business, and environmental care. The participants were guaranteed anonymity, and allowed to discontinue the study at any time. All participants indicated informed consent electronically, in accordance with the declaration of Helsinki. The study was approved by the Vice-Rector of Research at The Norwegian School of Economics.

**FIGURE 2:**
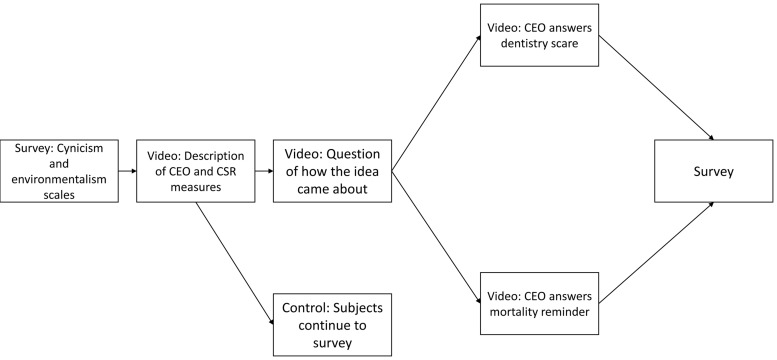
**Experimental procedure: Experiment 1**.

Participants were randomly assigned to one of two experimental groups and one passive control group. The participants assembled in different classrooms. Before the experiment started, they were given instructions on how to respond during the experiment via their smartphones, tablets, or computers. The participants were furthermore instructed to give responses individually, and maintain silence throughout the experiment. The participants first responded to Kanter and Mirvis’ (1989) dispositional cynicism scale, and Milfont and Duckitt’s (2010) environmental attitude scale. They were then exposed to one of three different videos, as described in the section above. The video was displayed on a big screen in the classroom. Participants in the passive control condition (*N* = 27) saw the video wherein no justification was asked for, and went on to complete the survey. The second group saw the active control dentistry video (*N* = 29), and continued to give their responses on the outcome measures. The third group saw the mortality awareness video (*N* = 31), and continued to give their responses on the outcome measures. All participants had to view the entire designated video before they could move on in the experiment. **Figure 2** illustrates the experimental procedure.

### Dependent Measures

Having seen their respective video, all participants were instructed to complete a survey, detailing their attitudes toward the decision and the decision maker, as well as perceived communicative intention, and motivational attributions pertaining to the decision (see Appendix for a complete overview of all). All outcome measures were entered into a principle components factor analysis, with direct oblim rotation. The analyses extracted four factors with an eigenvalue above 1.00. According to Singh (1991), deviations from the normative use of one as eigenvalue cut-off score is permissible in cases where adherence to the norm would produce redundancy in constructs. Using scree plot analyses of the eigenvalues (see Hair et al., 1998) a drop in eigenvalue between the fifth and sixth factors was identified. This was consistent with the expected factor structure. As the factor analysis was used mainly to investigate the internal consistency of my measures, we applied similar criteria to Rust et al. (2004) (parsimony, managerial usefulness, and psychological meaningfulness) and applied a five factor structure in these analyses. **Table 1** displays the measurement model.

**Table 1:** Measurement model in Experiment 1.

Item:	Mean	*SD*	Factor loadings				
			1	2	3	4	5
Like decision maker	4,81	1,18				-0.595	
Endorse decision maker	4,31	1,55				-0.671	
Decision maker is professional	4,95	1,36				-0.918	
Decision maker is competent	4,77	1,24				-0.935	
Moral motivation	4,92	1,42	0.719				
*… they have a long-term interest in society*	3,39	0.94	0.889				
*… they believe in environmental care*	3,76	0.96	0.861				
*… they are trying to give back to society*	3,07	1,02	0.817				
*… their customers expect them to do this*	3,43	1,14		0.922			
*… society in general expects them to do this*	3,56	0.973		0.833			
*… they will retain more customers by doing this*	3,38	0.94		0.721			
Attitudinal persuasion knowledge	4,06	0.89			0.844		
Behavioral persuasion knowledge	3,93	0.89			0.890		
Willingness to pay	4,38	0.78					0.944

Liking decision maker, perceiving decision maker as professional, perceiving decision maker as competent, and willingness to endorse decision maker as a member on an advisory board on ethical investment were all measured on seven-point Likert scales. These items formed the factor *evaluation of decision maker* (factor 4 in **Table 1**) (Cronbachs α = 0.84). A single item measuring liking decision had to be removed due to multicollinearity. The CSR attribution scale from Groza et al. (2011) was also deployed, but due to cross-loading, only six of the nine items were included in the analysis. The three items measuring value-driven attribution were combined with the single item measuring perceived moral motivation to form the factor *value-based attributions* (factor 1 in **Table 1**) (Cronbachs α = 0.84). The mean score on the three remaining attribution items was labeled *extrinsic attribution* (factor 2 in **Table 1**) (Cronbachs α = 0.72). The concept *persuasion knowledge* refers to the extent to which a person feels that the communicative intention of another person is to manipulate or persuade them. In this experiment, persuasion knowledge refers to the extent to which participants felt that the CEO had such a communicative intention, and was measured with two items (factor 3 in **Table 1**). One item assessed attitudinal persuasion knowledge (i.e., “*the CEO is attempting to change my attitudes*”) and another item assessed behavioral persuasion knowledge (i.e., “*the CEO is attempting to influence my future choices*”) (Cronbachs = 0.70). However, the measure was not used in the further analysis. Finally, participants were asked to indicate their willingness to pay in reference to a normal price for an equivalent room at an equivalent hotel, given their new information (factor 5 in **Table 1**). The participants indicated their *willingness to pay* on a seven-point scale, with zero percent as center value, and 10% increases or decreases in price in each direction, with 30% more and 30% less as extreme values. All standardized scales used in this study were translated to Norwegian. The following process assured the quality of the translation: first, three Ph.D. students working separately produced a translation of each item. Secondly, three bilingual professors at the department of language choose which translation was correct. The professors provided their votes separately. Across all items, the professors were unanimous in all but two cases, in which the version with two out of three votes was selected. This translation procedure is very similar to the one recommended by Douglas and Craig (2007).

### Results

**FIGURE 3:**
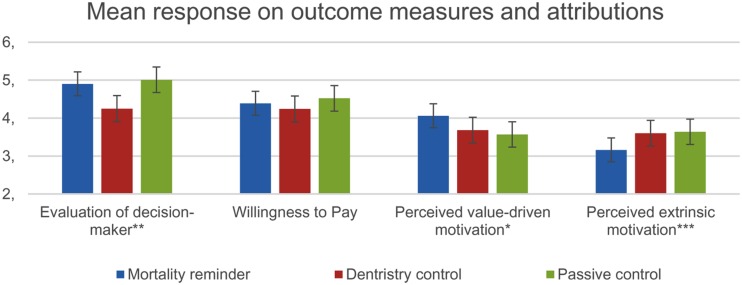
**Mean evaluation between groups**. ^∗^ Difference between mortality awareness and passive control is significant at *p* < 0.05. ^∗∗^ Difference between mortality awareness and active control is significant at *p* < 0.05. ^∗∗∗^ Differences between mortality awareness and both control groups are significant at *p* < 0.05.

Correlational findings from this experiment were in line with past research that has shown that motivational attribution is a crucial factor for positive evaluation in CSR communication (Du et al., 2010; Groza et al., 2011; de Vries et al., 2013). A high score on the value-based attribution measure was positively associated with liking the decision (*r* = 0.51, *p* < 0.01), and a positive overall evaluation of the decision maker (*r* = 0.58, *p* < 0.01). The two first hypotheses predicted between-groups differences in how the CEO would be perceived and how her decision would be attributed. In order to test the hypotheses, we conducted a series of ANOVAs where condition (mortality awareness vs. active control vs. passive control) was always the between-subject variable, and where the dependent variables were evaluation of decision maker, willingness to pay, extrinsic attribution, and value-based attribution, respectively. As we were only seeking to address the question of how mortality awareness can produce differences in evaluation and attribution, we performed planned contrasts of [mortality awareness] vs. [active control + passive control]. We also performed planned contrasts between the two active conditions, i.e., [active control] vs. [mortality awareness]. According to Hypothesis 1, the CEO would be perceived more favorably by participants in the mortality awareness condition than by participants in the two control groups. The results revealed statistically significant differences between group means [*F*(2,84) = 4.13, *p* = 0.02]. The planned contrast tests indicated that the CEO was given a more positive evaluation by participants in the mortality awareness condition compared to the active control condition CEO [*t*(83) = -2.16, *p* = 0.02, *d* = 0.57, 95% CI from 0.30 to 0.86], but not compared to both control conditions combined [*t*(83) = 1.11, *p* = 0.69, *d* = 0.25, 95% CI from -0.48 to -0.02]. Hypothesis 1a was thus only partially supported. We further predicted that the mortality salient CEO would produce a higher willingness to pay than the control conditions. The ANOVA did not provide support for this prediction [*F*(2,84) = 0.88, *p* = 0.42], and the planned contrast tests demonstrated no significant differences in willingness to pay. Hypothesis 2 stated that the CEO whose justification involved mortality awareness would produce more value-based attributions, and less attribution to extrinsic motivations. The ANOVA failed to demonstrate significant between groups differences [*F*(2,84) = 2.13, *p* = 0.09]. However, the planned contrast tests indicated that the mortality salient CEO came across as significantly more motivated by intrinsic values, compared with the two control groups combined [*t*(84) = 2.18, *p* = 0.03, *d* = 0.49, 95% CI from 0.31 to 0.68], but not when compared solely with the active control group [*t*(84) = -1.66, *p* = 0.10, *d* = 0.43, 95% CI from 0.23 to.61]. Hypothesis 2a thus only received partial support. Finally, the groups were found to differ significantly, in line with the hypothesis, with regards to attributing the decision to extrinsic motivation [*F*(2,84) = 4.20, *p* = 0.02]. The planned contrast test showed that the mortality aware CEO came across as significantly less motivated by extrinsic factors, compared to the two control conditions combined [*t*(84) = -2.89, *p* < 0.01, *d* = -0.63, 95% CI from -0.82 to -0.50], and compared to only active control [*t*(84) = 2.42, *p* = 0.01, *d* = -0.53, 95% CI from -0.82 to -0.43]. This finding supported Hypothesis 2b. **Figure 3** displays the mean evaluations between the groups in perceptual outcomes and motivational attribution.

**FIGURE 4:**
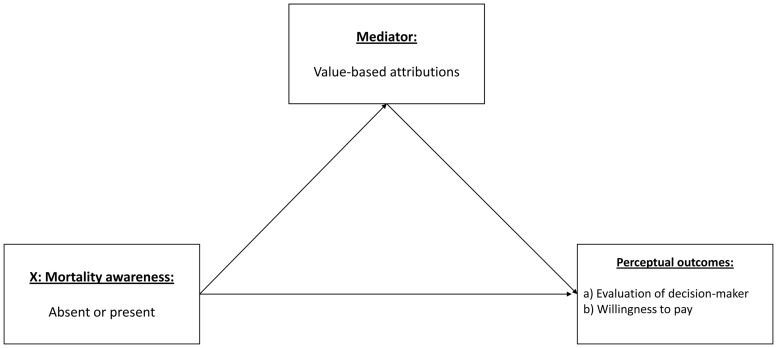
**Predicted mediated relationship**.

In order to test Hypothesis 3, two simple mediation analyses were conducted. Across the analysis of differences between the groups, the active control group and passive control group did not differ significantly on any dispositional measures or outcome measure except evaluation of decision maker. While hypotheses 1 and 2 predict direct effects of mortality awareness compared to both passive and active control stimuli, hypotheses 3a and 3b represent attempts at exploring the mediating factors that are affected by the presence or absence of mortality awareness. As such, and in order to increase the validity and power of the mediation analysis necessitated by Hypothesis 3, the results from the active and passive control groups were combined into a single group. The tested models are illustrated in **Figure 4**.

**FIGURE 5:**
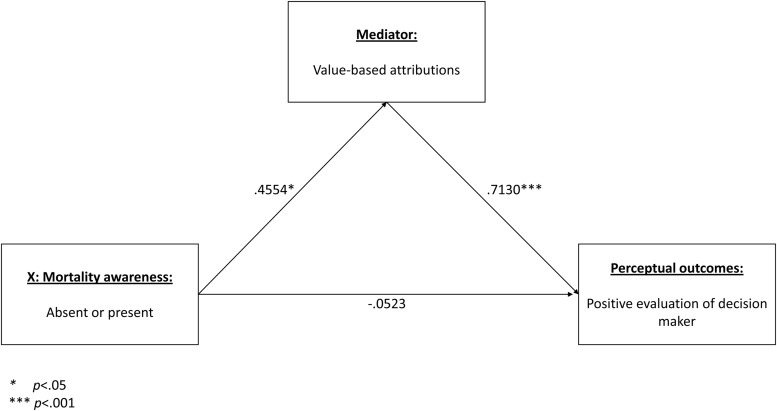
**Indirect effect of mortality awareness on positive evaluation of decision maker, via increased value-based attributions**.

We employed Preacher and Hayes’s (2004, 2008) PROCESS macro for SPSS. The proposed mediation models revealed no significant direct effect on any of the perceptual outcome measures. Although the direct effects are absent, it is still possible and useful to test and report the indirect relationships in the proposed model (see Hayes, 2009). In such analyses, where a direct effect between X and Y is absent, the proposed mediators are better referred to as indirect relationships, rather than mediated relationships (Mathieu and Taylor, 2006). The first predicted relationship was between presence or absence of mortality awareness, value-based attribution, and evaluation of decision maker. The analysis revealed a significant indirect effect. A bias-corrected bootstrap confidence interval for the indirect effect based on 10,000 bootstrap samples was entirely above zero (from 0.0524 to 0.6469). The coefficients revealed that presence of mortality awareness led to significantly higher levels of value-based attribution, which in turn was significantly positively associated with positive evaluation of decision maker. Hypothesis 3a was thus supported. **Figure 5** displays the model, with coefficients.

**FIGURE 6:**
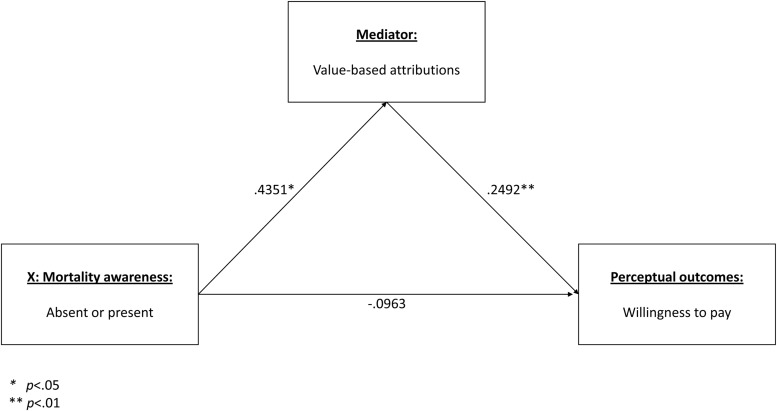
**Indirect effect of mortality awareness on willingness to pay, via increased value-based attributions**.

In order to test Hypothesis 3b, the same analysis was repeated, but with willingness to pay as outcome measure. Once again, the indirect effect proved significant, with confidence intervals entirely above zero (from 0.0249 to 0.2887). As predicted, the coefficients revealed that the presence of mortality awareness led to higher levels of value-based attribution, which in turn was found to be significantly positively related to higher willingness to pay. These findings underscore the importance of value-based attribution as antecedent to favorable outcomes in strategic CSR communication. In as much as the predicted mechanisms were proven significant, Hypothesis 3 is supported. **Figure 6** displays the mediation model with corresponding coefficients.

### Discussion

The results from Experiment 1 demonstrated that the decision maker who had recently suffered a mortality reminder was seen as more motivated by moral values, and significantly less by extrinsic or instrumental factors. Furthermore, the support of Hypothesis 3 demonstrates that this difference in attribution is a relevant factor when participants evaluate the decision maker, and when they determine their willingness to pay. However, having recently been reminded of her own mortality did not change evaluation of the decision maker or willingness to pay directly. Nevertheless, the results offer support for the notion that ambiguously motivated decisions made by a decision maker who appears aware of her own mortality is seen as less driven by extrinsic motivation. The first experiment thus demonstrates that the identified main effect of mortality primes is also relevant in social perception: people expect a decision maker to be less motivated by extrinsic factors when she has recently been exposed to a mortality reminder. This result is allegorical to the general finding from TMT, which states that people tend to trivialize the importance of extrinsic pursuits after a mortality prime (Burke et al., 2010). However, the TMT literature suggests that the relationship between mortality salience and motivation for extrinsic pursuits is moderated by the personal priority placed on the extrinsic pursuit in question (Kosloff and Greenberg, 2009). The moderated relationship states that mortality primes remove motivation for low-priority extrinsic pursuits, but increases the motivation for high-priority extrinsic pursuits (Arndt et al., 2004). As the importance of this moderator has been readily demonstrated within the TMT tradition, the aim of the second experiment in this paper was to explore whether the same moderated relationship is relevant in perception of others.

## Experiment 2

The aim of Experiment 2 was to explore the extent to which mortality awareness may serve as an indicator of value-driven motivation in cases where the observed decision maker places a high importance on the attainment of financial goals. As mentioned, TMT research indicates that mortality primes mainly tend to amplify investments in whatever themes are personally and/or culturally important to the decision maker (Burke et al., 2010). Building on the assumption that people’s perceptions of the motivation of other people largely parallels their own motivational processes (Reeder and Trafimow, 2005), it is reasonable to hypothesize that their lay-theories about other people’s motivation under mortality awareness would also encompass this general amplification tendency. If people perceive others in line with relevant results from TMT research, an ambiguously motivated decision made by a recently mortality reminded decision maker who is known to place a high priority on financial goal attainment should generate the opposite attributional pattern from the one demonstrated in the first experiment. More specifically, if the findings from Kosloff and Greenberg (2009) translate into perception of others, the decision maker who is both acutely aware of her mortality, and known for placing a high priority on financial goal attainment, should be interpreted as being more motivated by the extrinsic gains associated with the decision, and less by the claimed moral or value-based motivation for the decision. The first hypothesis was thus:

H1. In cases where the observed decision maker is known for placing a high personal priority on financial goal attainment, reporting that a mortality reminder preceded the decision to implement environmentally and financially beneficial policies will lead to more attribution to financial motivation.

Past research on CSR perception and motivational attribution has demonstrated that endeavors that are perceived as self-serving in a financial sense are often perceived as less motivated by morality or intrinsic values (Groza et al., 2011; de Vries et al., 2013). We therefore further predicted that the recently mortality reminded decision maker would be perceived as less motivated by intrinsic values and morality. The second hypothesis was therefore:

H2. In cases where the observed decision maker is known for placing a high personal priority on financial goal attainment, reporting that a mortality reminder preceded the decision to implement environmentally and financially beneficial policies will lead to less attribution to moral motivation.

Moral psychology has demonstrated that inferences of motivation are crucial when people make judgments about how morally praiseworthy a behavior is (Fedotova et al., 2011; Gray et al., 2012). Similarly, CSR research has demonstrated that the perceived motivation behind environmental policies influence peoples’ general perception of those policies, and the companies that employ them (Windsor, 2013; de Vries et al., 2013; Olsen et al., 2014). Our last prediction was therefore that the changes in motivational attribution produced by the stated mortality reminder would produce overall worse evaluation of the decision and decision maker. The third and final hypothesis was thus:

H3. In cases where the observed decision maker is known for placing a high personal priority on financial goal attainment, reporting that a mortality reminder preceded the decision to implement environmentally and financially beneficial policies will lead more negative evaluation of (a) the decision and (b) the decision maker.

### Procedure, Materials, and Sample

One hundred and eighty undergraduate psychology students (140 female, mean age 20) were recruited to participate in the experiment. Participation was voluntary and not compensated. Before the experiment, participants were told that the experiment would be about communication, business, and environmental care. The participants were guaranteed anonymity, and allowed to discontinue the study at any time. All participants indicated informed consent electronically, in accordance with the declaration of Helsinki. The study was approved by the Vice-Rector of Research at The Norwegian School of Economics.

Again, a between-groups design was applied. Groups to witch students had been randomly assigned were randomly assigned to the different conditions, i.e., the passive control group, the dentistry control group, or the mortality awareness group. The experiment was conducted on three consecutive days. The experimental procedure was identical to that of the first experiment. The participants first indicated their responses to the dispositional and attitudinal measures. After all participants had completed these surveys, they were exposed to one of the three different video stimuli. The only difference between the stimuli used in this experiment, relative to the stimuli used in the first experiment, was that the text part of the video contained one additional piece of information, regarding the emphasis placed on financial pursuits by the observed decision maker. The text in the videos in the second experiment read: *“Ellen Hansen is the CEO of a large Nordic hotel chain. She has always had an intense desire to make her hotel chain the most profitable in Europe. One of her biggest life-time goals has been to ensure that the company generates over 100 million NOK in profits in one fiscal year.* [new paragraph] *Ellen has just approved a new plan to make the hotels more environmentally friendly. Among the measures are reducing the size of plates and glasses at the breakfast buffet with 20%, in order to reduce waste of food. Furthermore, the hotels have been equipped with water-saving showers. Additionally, the hotels have adopted a more restrictive policy on changing of towels and linens on shorter stays.* [new paragraph] *In an interview, Ellen explained the background for the decision:”* The passive control condition participants (*N* = 57) saw the video wherein no justification is asked for, and went on to complete the survey. The second group saw the active control dentistry video (*N* = 55), and continued to give their responses on the outcome measures. The third group saw the mortality salient video (*N* = 68), and continued to give their responses on the outcome measures. All participants had to view the entire designated video before they could move on in the experiment.

### Dependent Measures

Dependent measures were identical to those used in Experiment 1. Additionally, in order to better capture the motivational attributions of interest in this study, the measurement model in the second experiment was supplemented with more attribution measures. The additional attribution measures were all in the form of statements, with which the participants indicated their agreement on seven-point Lickert scales. The statements all started with “*It seems like an important goal with these measures is*”. The proposed perceived goals were; *please customers, save money, ensure financial solidity*, and *reduced climate change*. We also added new items assessing support for the decision itself, as the single item used in Experiment 1 had to be removed from analysis due to cross-loading. The new items measures support for decision from both a consumer and employee perspective, principled support, an overall support item, and a reversed support item. All outcome measures were initially entered into a Principle Components factor analysis, with direct oblim rotation. The items measuring level of endorsement for the CEO to serve at ethics committee, as well as the item measuring willingness to pay had to be removed due to issues with multicollinearity and cross-loading. **Table 2** displays the final measurement model used in the experiment.

**Table 2:** Measurement model in Experiment 2.

Item:	Mean	*SD*	Factor loadings						
			1	2	3	4	5	6	7
As an employee, support for decision	6,28	1,1	-0.745						
As a customer, support for decision	5,93	1,27	-0.669						
The decision is good	4,33	0.761	-0.755						
Principled support for decision	4,54	0.712	-0.952						
Disagree with decision (reversed)	4,52	0.758	0.886						
Like decision maker	4,49	1,48		0.650					
Decision maker seems professional	4,85	1,49		0.950					
Decision maker seems competent	5,00	1,37		0.784					
Perceived moral motivation	5,02	1,49			0.666				
Goal appears to be reduced climate change	3,84	1,05			0.670				
*…they have a long term interest in society*	3,55	1,06			0.740				
*… they believe in environmental care*	3,77	1,04			0.886				
*… they are trying to give back to society*	3,15	1,05			0.795				
*… they think their customers expect them to do this*	3,17	1,15				0.816			
*… they think society in general expects them to do this*	3,48	1,13				0.929			
*… they think their owners and other stakeholders expect them to do this*	3,12	1,05				0.815			
*… they think they will retain more customers by doing this*	3,60	0.992					-0.902		
*… they think they will get more customers by doing this*	3,62	1,06					-0.856		
Goal appears to be to please customers	3,71	1,02					-0.564		
Perceived financial motivation	4,43	1,70						0.742	
Goal appears to be to save money	3,31	1,14						0.878	
Goal appears to be to ensure financial solidity	3,37	1,04						0.839	
*… they think they will earn/save more money by doing this*	3,93	1,03						0.825	
Attitudinal persuasion knowledge	4,02	0.948							0.898
Behavioral persuasion knowledge	3,86	1,04							0.892

The results from the factor analysis revealed seven distinct factors, consistent with the expected factor structure. The factors are numbered in accordance with their position in **Table 2**. The first factor was labeled *support for decision* (1) (Cronbachs α = 0.86). The second factor was labeled *evaluation of decision maker* (2) (Cronbachs α = 0.83). The third factor was labeled *value-based attribution* (3) (Cronbachs α= 0.88). This factor consisted of the same four items that made up the same factor in Experiment 1, plus the addition of one item; *the purpose appears to be reduced climate change*. The fourth factor was labeled *reactive attribution* (4), meaning that the measures are seen more as motivated by external expectations (see Groza et al., 2011) (Cronbachs α = 0.86). The sixth factor was labeled *financial attribution* (6) (Cronbachs α = 0.85). The fifth and seventh factor were not utilized in further analysis.

### Results

**FIGURE 7:**
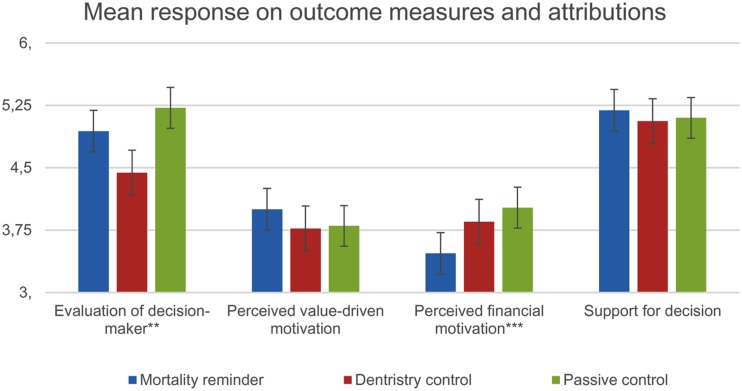
**Mean evaluation between groups, when decision maker is known for prioritizing financial goal attainment.** ^∗^ Difference between mortality awareness and passive control is significant at *p* < 0.05. ^∗∗^ Difference between mortality awareness and active control is significant at *p* < 0.05. ^∗∗∗^ Differences between mortality awareness and both control groups are significant at *p* < 0.05.

The three hypotheses predicted between-groups differences in how the CEO would be perceived and how her decision would be attributed. In order to test the hypotheses, we conducted a series of ANOVAs where condition (mortality awareness vs. active control vs. passive control) was always the between-subject variable, and where the dependent variables were evaluation of decision maker, support for decision, financial attribution, value-based attribution, and reactive attribution, respectively. As we were only seeking to address the question of how mortality awareness can produce differences in evaluation and attribution, we performed planned contrasts of [mortality awareness] vs. [active control + passive control]. We also performed planned contrasts between the two active conditions, i.e., [active control] vs. [mortality awareness]. The degree of financial attribution evoked differed significantly between the groups, but not in line with Hypothesis 1 [*F*(2,177) = 4.81, *p* < 0.01]. The planned contrast test showed that the mortality aware CEO induced lower levels of financial attribution than the CEO presented in the two control conditions combined [*t*(177) = -2.96, *p* < 0.01, *d* = -0.45, 95% CI from -0.61 to -0.31], and active control in isolation [*t*(177) = 2.05, *p* = 0.042, *d* = -0.36, 95% CI from -0.55 to -0.17]. These findings failed to provide support for the first hypothesis, which stated that the decision made under mortality awareness would be more attributed to financial motivation. Furthermore, the analyses showed no significant group differences in value-based attribution [*F*(2,172) = 1.00, *p* = 0.37]. The planned contrast test revealed no significant difference between the mortality awareness condition and the two controls combined [*t*(172) = 1.41, *p* = 0.16, *d* = 0.22, 95% CI from 0.09 to 0.36], and no difference when compared only against active control [*t*(172) = -1.28 *p* = 0.20, *d* = 0.24, 95% CI from 0.08 to 0.40]. This finding did not offer support for the Hypothesis 2, which predicted that the mortality awareness CEO would produce less value-driven attributions, compared to the control conditions. Finally, the ANOVA revealed no significant group differences in support for the decision [*F*(2,177) = 0.48, *p* = 0.62]. Neither of the planned contrasts showed significant differences. However, the analysis did reveal a significant difference in evaluation of decision maker [*F*(2,177) = 5.88, *p* < 0.01]. The planned contrast test showed a non-significant difference between the mortality awareness condition and the two controls combined [*t*(177) = 0.55, *p* = 0.58, *d* = 0.08, 95% CI from -0.11 to 0.26]. However, there were significant differences between the mortality awareness CEO and the active control, in terms of evaluation of decision maker [*t*(177) = -2.23, *p* = 0.03, *d* = 0.38, 95% CI from 0.16 to 0.61]. These findings failed to provide support for the third hypothesis, which stated that the mortality awareness CEO would produce the lowest support for her decision, and lowest levels of evaluation of decision maker. Aside from testing the hypotheses, we also explored between groups differences in reactive attributions. The groups were found to differ significantly in terms of reactive attributions [*F*(2,177) = 3.10, *p* = 0.04]. The actions of the mortality aware CEO were perceived to a lesser degree to be a reaction to external expectations, compared with the two control conditions combined [*t*(177) = -2.56, *p* = 0.01, *d* = -0.39, 95% CI from -0.54 to -0.26], and compared only to the active control condition [*t*(177) = 2.23, *p* = 0.027, *d* = -0.42, 95% CI from-0.59 to -0.26]. **Figure 7** displays the mean evaluations and attributions between groups.

### Discussion

According to Kosloff and Greenberg (2009), as well as Arndt et al. (2004), the decision maker portrayed in this experiment should be expected to be more motivated by financial goal attainment as a consequence of her recent mortality reminder, combined with her life-long ambitions of financial goal attainment. However, the results clearly indicate that participants did not perceive the CEO in this manner. Even though TMT studies have demonstrated the moderating role of personal importance of financial pursuits, the present results indicate that people’s attributions of others are not sensitive to the presence of this moderator. The lack of support for any of the hypotheses, can be interpreted in terms of the observed decision makers’ priority of financial gains being less relevant in social perception and attribution. Much like in Experiment 1, the decision maker who explained that she had recently been exposed to a real life mortality reminder was perceived as less motivated by financial gain and external expectations, even though it was clearly stated that financial goal attainment had long been a guiding goal for the decision maker in question. Put in less technical terms, the participants’ attributions fell more along the lines of what one would predict after reading about Dickens Ebenezer Scrooge, and less in line with the predictions one would make after reading TMT research on how financially oriented people actually tend to respond to mortality reminders. The only notable difference between the results from Experiments 1 and 2 was that the former produced a significant difference in value-based attributions, while the latter did not. It would thus seem that mortality awareness increases value-based attribution in cases where the decision maker is unknown, but not in cases where the decision maker is known for placing a high priority on financial goal attainment. However, external and financial attributions were reduced as a consequence of mortality awareness, regardless of the personal priorities of the observed decision maker.

## General Discussion

Taken together, the results confirm that communicating mortality awareness when justifying ambiguously motivated CSR initiatives alleviates suspicions of extrinsic motivation, and may leave observers with a stronger perception that the decision is motivated by sincerely held values. This attributional effect holds true even in cases where the observed decision maker is known to place a high priority on the pursuit of financial goal attainment.

Both experiments demonstrated that ambiguously motivated CSR measures are met with more favorable attribution when the CEO explains that the motivation came after a mortality reminder. There are several direct implications from this result. Firstly, it demonstrates that the same set of CSR measures can induce very different motivational attributions among observers, based on how those CSR measures are presented. Secondly, the results indicate that people suspect behaviors to be less motivated by extrinsic and financial factors, when the person they evaluate attributes her decision to a recent mortality reminder. This change in attribution is likely not due to pity alone, as the active control condition, which presumably also induces pity, produced attributions more similar to that of the passive control condition than that of the mortality awareness condition. Furthermore, the improved motivational attribution is demonstrated to be indirectly associated with evaluation of decision maker and willingness to pay. This finding highlights the importance of perceived sincerity of motives when communicating CSR policies, which resonates concordantly with other findings in CSR communication (Du et al., 2010; Groza et al., 2011; de Vries et al., 2013).

The results offer some careful practical implications. Motivational ambiguity is ubiquitous in both general management and marketing. The main result from this study is that mortality awareness can function as a factor that alleviates some of that ambiguity, and induces a sense of sincerity on behalf of the observed decision maker. Presumably, other ways of conveying mortality awareness, other than recapping one’s own recent mortality reminders, can produce similar benefits. Invoking a life-death narrative when communicating ambiguously motivated CSR measures might produce similar results, in that peoples tendency to attribute the initiatives to extrinsic factors decreases. The results further indicate that people expect a drop in the priority of financial gains as a consequence of mortality salient experiences. This finding gives further practical implications. When faced with suspicion of being motivated by greed, rather than virtue, invoking a mortality salient narrative behind one’s decisions can give an indication of authenticity, thus reducing cynical attributions and creating more engagement and approval for the decision.

The experiment made use of vivid and realistic video-stimuli, in order to increase ecological validity. Nevertheless, we want to highlight some potential limitations. As is the case for almost all lab-experiments in social sciences, the results stem from hypothetical scenarios, and are therefore free from circumstantial factors that may be crucial to any real-life scenario involving the role of mortality primes in eliciting different attributions. Furthermore, the distinction between the active control condition (dentistry) and experimental manipulation (mortality reminder) has been subject to some criticism (Burke et al., 2010). TMT research has typically suggested that the threat of mortality produces qualitatively different consequences than similar non-mortality control primes. Indeed, the premises and conclusion of this paper partially rely on the same assertion. However, it cannot be definitely concluded that the distinction between mortality primes and other negative events are qualitative, and not quantitative. If the only real difference between the active control and the mortality awareness manipulation is the level of pity induced in the participants, a theoretical implication would be that sufficiently elevated levels of pity, not perceived mortality awareness, might produce a similar effect. In both the active control condition and mortality awareness condition, the CEO links her account of health problems to the CSR decision by the sentence fragment “[…] it got me thinking about what really matters in life”. This sentence was necessary in order for the CEO’s statements to be meaningful in their given context. However, we cannot rule out that this statement comes across as more plausible and sincere when stated as a consequence to a potentially lethal health-scare, than a non-lethal health scare. We recommend that future studies of perceptions of motivation under mortality awareness should attempt to continue to explore these processes in ecologically valid manners, while attempting to keep the experimental condition and the active control condition as similar as possible. The presented research was primarily concerned with between-groups differences in evaluation and attribution, based on the CEO’s reported mortality awareness. In order to explore this effect, other factors pertaining to the CEO were held constant across conditions. There is thus a possibility that the reported tendencies are the result of interaction effects between mortality awareness and unique factors pertaining to the CEO, such as gender, age, physical appearance or other visible factors.

The results offer some further directions for future research. First, the degree of liking the decision to implement the CSR initiatives, evaluation of decision maker and willingness to pay, did not differ between the groups in any of the experiments, even though the attributional patterns differed significantly between the groups. This finding goes against the importance placed on motivational attribution in past research, both in CSR communication (see Yoon et al., 2006; Du et al., 2010; Groza et al., 2011; McShane and Cunningham, 2012; de Vries et al., 2013), and in moral psychology (see Fedotova et al., 2011; Gray et al., 2012). A possible explanation for why the changes is motivational attribution were not accompanied by direct changes in evaluation of decision maker, support for decision or willingness to pay, may be that the described scenario entailed removing hedonic value for the customer. Past research has indicated that consumers often dislike CSR measures that impedes the organizations ability to deliver value to the consumer (Sen and Bhattacharya, 2001; Luo and Bhattacharya, 2006). As such, it may be that the participants responses to the question of whether or not they liked the decision was overly focused on the removal of hedonic value, making the issue of motivational attribution less salient. Other mediating variables, not measured in the experiments, may also have influenced the outcomes. However, these are merely speculative interpretations, and more research is needed in order to better understand the antecedents of consumer support for CSR measures.

## Conclusion

The problem of negative motivational attribution hinders the endorsement of effective CSR measures that can mitigate climate change. The presented research explored the role of mortality awareness on attribution of environmentally friendly behaviors. The first experiment demonstrated that when a CEO implements environmentally friendly corporate measures after a mortality reminder, her motivation for doing so was perceived as more value-oriented, meaning that it was attributed more to intrinsic values, and less to extrinsic factors. This attributional pattern was indirectly linked to an increase in positive evaluation of decision maker and increased willingness to pay, even though the CSR measures entailed removing hedonic value at the customers’ expense. The second experiment demonstrated that the reduction in extrinsic and financial attribution observed in the first experiment persisted, also when the decision maker was known for placing a high priority on financial goal attainment.

## Author Contributions

Both authors have contributed substantially in all parts of the research process.

## Conflict of Interest Statement

The authors declare that the research was conducted in the absence of any commercial or financial relationships that could be construed as a potential conflict of interest. The reviewer JPF and handling Editor declared their shared affiliation, and the handling Editor states that the process nevertheless met the standards of a fair and objective review.
